# *MarLe*: Markerless estimation of head pose for navigated transcranial magnetic stimulation

**DOI:** 10.1007/s13246-023-01263-2

**Published:** 2023-05-11

**Authors:** Renan H. Matsuda, Victor H. Souza, Petrus N. Kirsten, Risto J. Ilmoniemi, Oswaldo Baffa

**Affiliations:** 1 Department of Physics, Faculty of Philosophy Sciences and Letters of Ribeirão Preto, University of São Paulo, Av. Bandeirantes, Ribeirão Preto, 3900, 14040-901 SP Brazil; 2 Department of Neuroscience and Biomedical Engineering, Aalto University School of Science, Rakentajanaukio 2, Espoo, 02150 Finland; 3 School of Physiotherapy, Federal University of Juiz de Fora, Juiz de Fora – MG, Cascatinha, Brazil

**Keywords:** Neuronavigation, Transcranial magnetic stimulation, Markerless tracker, Face detection, Coregistration

## Abstract

Navigated transcranial magnetic stimulation (nTMS) is a valuable tool for non-invasive brain stimulation. Currently, nTMS requires fixing of markers on the patient’s head. Head marker displacements lead to changes in coil placement and brain stimulation inaccuracy. A markerless neuronavigation method is needed to increase the reliability of nTMS and simplify the nTMS protocol. In this study, we introduce and release MarLe, a Python markerless head tracker neuronavigation software for TMS. This novel software uses computer-vision techniques combined with low-cost cameras to estimate the head pose for neuronavigation. A coregistration algorithm, based on a closed-form solution, was designed to track the patient’s head and the TMS coil referenced to the individual’s brain image. We show that MarLe can estimate head pose based on real-time video processing. An intuitive pipeline was developed to connect the MarLe and nTMS neuronavigation software. MarLe achieved acceptable accuracy and stability in a mockup nTMS experiment. MarLe allows real-time tracking of the patient’s head without any markers. The combination of face detection and a coregistration algorithm can overcome nTMS head marker displacement concerns. MarLe can improve reliability, simplify, and reduce the protocol time of brain intervention techniques such as nTMS.

**Supplementary Information:**

The online version contains supplementary material available at 10.1007/s13246-023-01263-2.

## Introduction

Neuronavigation is crucial for transcranial magnetic stimulation (TMS) applications to increase the stimulation accuracy, reproducibility, and to reduce response variability [1–3]. Neuronavigation uses a tracking device to monitor the position and orientation of the TMS coil relative to the patient’s head. Tracking devices utilize markers or sensors fixed on the patient’s head and on the TMS coil for real-time position tracking [4]. However, marker fixation requires caution, as it must remain static during the entire treatment or experimental protocol. The accuracy of neuronavigation is compromised if the tracking markers move with respect to the brain. Small head-marker displacements can be left unnoticed by the operator, leading to critical inaccuracies in monitoring the TMS coil position relative to the brain [5].

In TMS, a strong current pulse applied into a coil placed on the scalp induces an electric field in the cortical tissue that can depolarize neurons [6]. Navigated TMS has been used in clinical applications for the treatment of neurological disorders [7, 8], in basic neuroscience research [9–11], and for mapping cortical functions prior neurosurgery [12], such as motor[13] and speech areas [14, 15]. However, slight changes in the coil placement may cause considerable unintended changes in the physiological responses [3, 5, 16, 17]. In conventional navigated TMS, head markers are susceptible to displacements during the coil positioning and can be challenging when combined with electroencephalography caps [18, 19]. Markerless tracking of the head position would increase the reliability of navigation and make the experimental procedure simpler and more accurate.

Face detection and recognition are well-established computer vision techniques. Existing algorithms can identify accurately non-face and face images and can discriminate different faces [20–22]. These techniques have been used for various applications such as surveillance systems and neuroscience studies based on facial expression and behavior recognition [23]. The combination of face detection and recognition enables real-time head pose estimation based on video recordings by facial landmark detection [24]. Automated head pose estimation open a possibility for the development of markerless neuronavigation. Studies have shown that markerless neuronavigation can aid in radiotherapy [25, 26], positron emission tomography [27] and neurosurgery [28]. However, the ensemble of markerless head pose estimation and TMS is a novel methodology.

To overcome the limitations imposed by physical head tracker displacements, we developed a markerless head tracker neuronavigation method for TMS, which we call *MarLe*. The main contributions of this study are: (1) a new method based on real-time head pose estimation that can be used with low-cost cameras and multiple tracking devices for nTMS. (2) We implemented and distributed our *MarLe* algorithm in our open-source neuronavigation system *InVesalius* [3]. (4) A full characterization of the accuracy and reliability of TMS navigation with markerless head pose estimation. (5) A streamlined process employing *MarLe*, which has the potential to enhance the ease of TMS targeting and reduce the duration of TMS procedures.

## Methods

The *MarLe* was implemented as a Python library distributed via cross-platform binary wheel files. Apart from conventional Python libraries, such as Numpy, the main dependencies of *MarLe* are:

1. OpenCV library [29] for processing, calibration, and camera communication
2. Dlib library [30] for the face detection and head-pose estimation

*MarLe* combines head-pose estimation from live video streaming with tool tracking. The live video can be provided by sufficiently high-definition video cameras while the tool tracking is optimally performed by dedicated spatial tracking devices. In this study, we validated and characterized the *MarLe* algorithm with three different video cameras, a built-in live stream video camera (resolution of 2048 × 1536 pixels; 20 frames per second (FPS)) in the video camera unit (VCU) Polaris Vega VT (Northern Digital Inc., Canada), and two low-cost webcams c270 (Logitech, Switzerland) (resolution of 1280 ⋅ 720 pixels; 30 FPS) and c920 (Logitech, Switzerland) (1920 ⋅ 1080 pixels; 30 FPS). Tracking of the TMS coil and the fiducials collection probe were performed with the Polaris Vega VT infrared camera and markers.

### Camera calibration

The camera calibration converts real-world three-dimensional (3D) position measurements to the camera’s coordinate system. Overall, the calibration estimates the intrinsic and extrinsic parameters of the camera. The intrinsic parameters are the camera geometry and lens distortion. The extrinsic parameters are the camera rotation and translation matrix relative to the tracked object. The intrinsic and extrinsic parameters are input arguments for *MarLe*. The camera calibration algorithm followed OpenCV checkerboard pattern calibration [31]. First, each camera captured 1000 checkerboard image samples from different viewpoints. Next, the camera parameters are computed based on Zhang’s [32] closed-form solution. Given the camera parameters, we found the coordinates of all checkerboard samples and we transformed them back to the two-dimensional (2D) camera coordinate system. Finally, we verified the camera calibration accuracy by computing the re-projection error. The re-projection error was calculated as the absolute norm between the transformed and the found checkerboard’s locations. The camera calibration algorithm was developed independently from *MarLe*.

### MarLe workflow

The workflow of *MarLe* has five steps:

1. Establish communication between the camera and the neuronavigation system;
2. Define the transformation matrix between the TMS coil tracking device and the camera;
3. Detect the face;
4. Transform head-pose estimation to the tracking device coordinate system;
5. Apply filtering to reduce jittering and measurement errors.

**Fig. 1:**
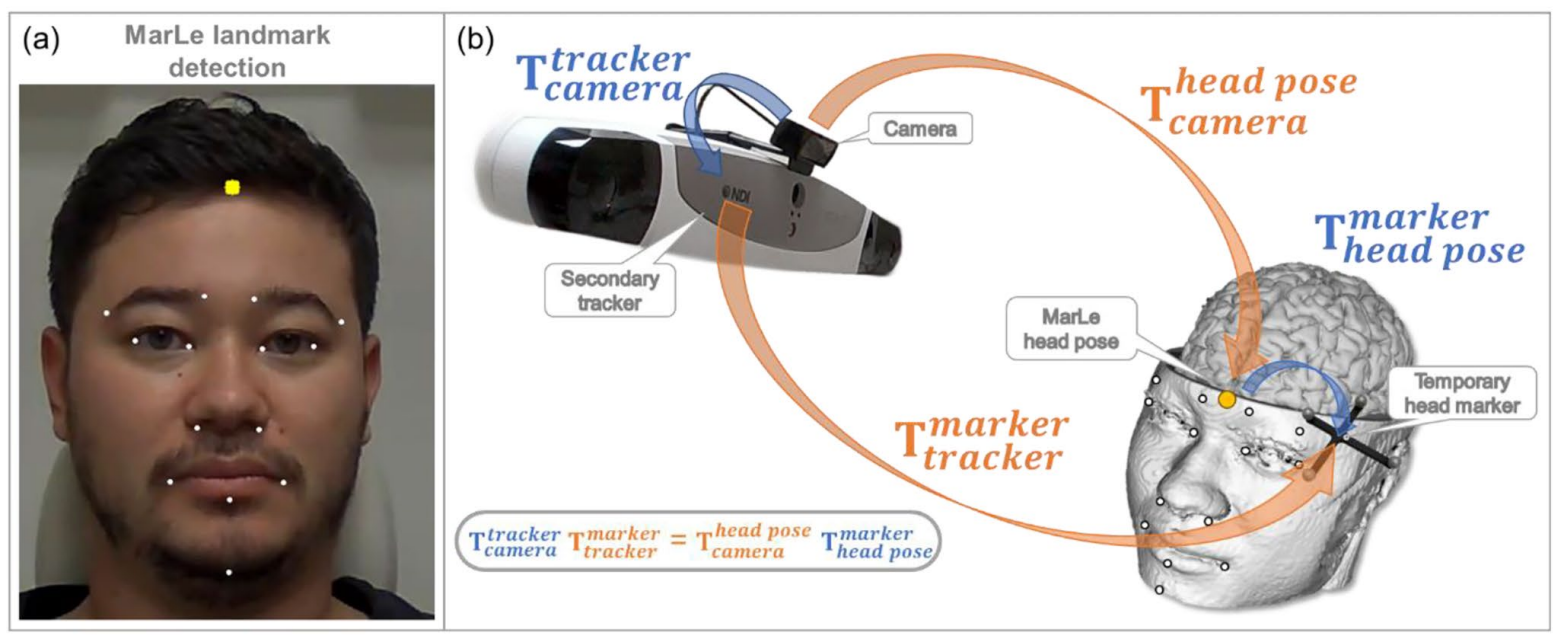
**a***MarLe* face detection of the first author of this paper; white dots represent the fitting of detected facial structures into the face video recordings. The yellow dot is the tracked coordinate of the head. **b** The transformations from the camera coordinate system to the coil tracker coordinate system are assumed to be constants, highlighted in blue as ${\mathbf{T}}_{camera}^{tracker}$ and ${\mathbf{T}}_{head pose}^{marker}$, under the assumption that the camera and the secondary tracker device are fixed. The orange transformations ${\mathbf{T}}_{tracker}^{marker}$ and ${\mathbf{T}}_{camera}^{head pose}$, represent homogeneous matrices from the camera to the tracking device and from the head pose to the head tracker, respectively

The OpenCV was used to establish the communication between the camera device and *MarLe* software. The user can set which camera will be used if multiple cameras are connected to the computer. The GStreamer backend, along OpenCV, was used to communicate with the VCU Vega VT. Once the camera communication is established, *MarLe* determines the position and orientation of the head. The *MarLe* algorithm employs the Dlib library along with a pre-trained network of facial landmarks to estimate the real-time 2D location of 68 facial landmarks [33]. The pre-trained network was generated based on the iBUG 300-W facial dataset [34]. Then, we use OpenCV, together with the camera calibration parameters, to find the projection of the 2D facial landmark to 3D. The projection was performed using a 3D anthropometric model proposed by Martins and Batista [24]. We selected 14 facial structures for the 3D fitting: inner and outer corners of both eyebrows, inner and outer corners of both eyes, right and left bottom corners of the nostrils, both labial commissure and the middle lower lip of the mouth, and finally the chin as shown in Fig. 1a.

We should note that *MarLe* provides only the markerless tracking of the head position, requiring a second device to track the TMS coil position. *MarLe* can be operated with any tracking device supported by the neuronavigation system. The *MarLe* and the secondary tracking device have different coordinates systems. Therefore, coregistration is required to operate both trackers in the same coordinate system. The coregistration consists in acquiring simultaneously the head pose in both coordinate systems. The *MarLe* collects the head pose based on the face detection and the secondary tracker collects the pose of a marker attached to the subject’s head, following the conventional neuronavigation workflow. We developed a graphical user interface to perform the coregistration between the two camera systems. The coregistration requires at least 500 head positions from each camera. The head pose estimation is transformed to the secondary tracking device coordinate system using a closed-form solution through the equation:

$${\mathbf{T}}_{camera}^{tracker} {\mathbf{T}}_{tracker}^{marker}= {\mathbf{T}}_{camera}^{head pose}{\mathbf{T}}_{head pose}^{marker}$$

where ${\mathbf{T}}_{camera}^{tracker}$ and ${\mathbf{T}}_{head pose}^{marker}$ are homogeneous transformation matrices from the camera to the tracking device and from the head pose to the head tracker, respectively. The ${\mathbf{T}}_{tracker}^{marker}$ and ${\mathbf{T}}_{camera}^{head pose}$ matrices are head-pose-paired measurements from both *MarLe* and the secondary tracking device. Once the coordinate system of the head-pose estimation and secondary tracking device are fixed, we compute the transformation matrices between the head-pose-paired measurements (${\mathbf{T}}_{tracker}^{marker}$ and ${\mathbf{T}}_{camera}^{head pose}$) using the least squares method. Figure 1b illustrates the transformation matrices. Once the coregistration is done, the head marker is removed from the subject’s head.

High-frequency noise oscillations, i.e., spatial jittering, in the head-pose measurements decrease the head-tracking accuracy [35]. The final step is the real-time smoothing filter to minimize the jittering of head-pose estimation. We implemented a Savitzky–Golay filter [36] based on the Python library SciPy [37]. This filter uses a convolution process to fit successive data sub-sets with a low-degree polynomial. The data subsets are given by fixed window size. A first-order polynomial filter was used with a window size of 5 frames. We also evaluated the response of Kalman and Grubbs filters, as described in the Supplementary Material. After comparing their performances, we chose the Savitzky–Golay filter due to its superior performance for attenuating jittering.

### Characterization

We characterized the stability of *MarLe* for different distances between the camera and the subject’s head, the jittering, and the accuracy in repositioning the TMS coil. To measure the stability, we used a frontal human face photo printed on an A4 office paper; the printed photo has a neutral facial expression and was printed with the original head size. We collect the head pose in three distances between the camera and the face picture: 100, 125, and 150 cm. Each acquisition was recorded for three minutes every two seconds for each condition. To evaluate the filter effect on the accuracy and stability, we recorded the head pose before and after applying the Savitzky–Golay filter. These measurements were repeated three times for each of the three cameras, Logitech c270, Logitech c920, and VCU from Polaris Vega VT. The jittering was estimated as the 95% interval (1.96 times the standard deviation) of each acquired coordinate. We used the same acquisition of the stability evaluation with 100-cm distance between camera and face, with filtering, and for the three cameras.

For validating the markerless navigation, we implemented *MarLe* on the neuronavigation software platform InVesalius [38]. InVesalius is an open-source and free software for navigated TMS. InVesalius supports multiple tracking devices. We characterized and demonstrated the *MarLe* with the Polaris Vega VT as the secondary tracking device. The accuracy of *MarLe* in repositioning the TMS coil was evaluated in a mockup experiment that followed a conventional TMS procedure [2] except that no TMS pulses were delivered to the subject. The study was approved by the local ethics committee of the University of São Paulo (CAAE: 54674416.9.0000.5407) in accordance with the Declaration of Helsinki.

**Fig. 2:**
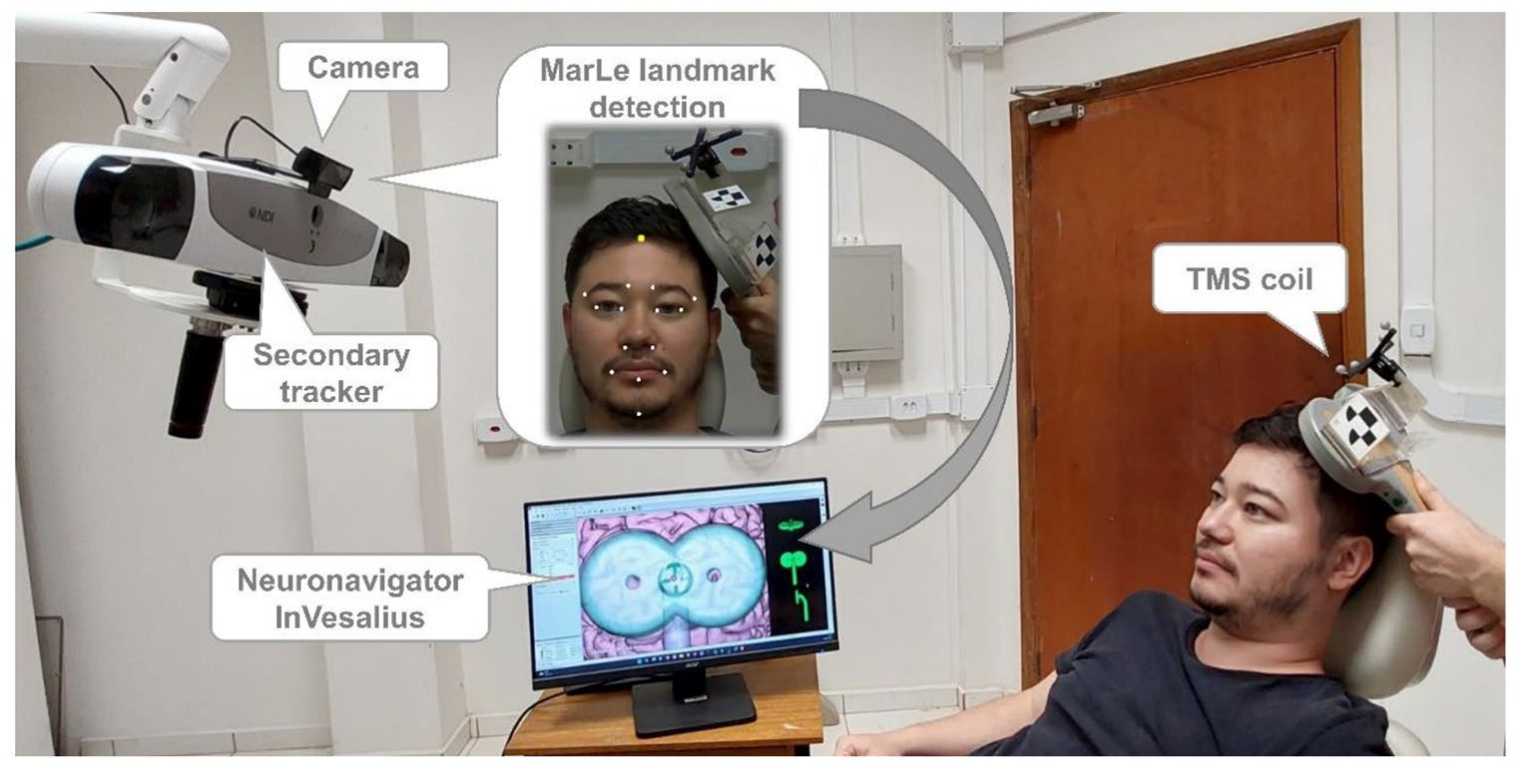
Experimental setup. The *MarLe* head pose estimation and the TMS coil pose are collected simultaneously. The head pose is transformed to the TMS coil tracker coordinate system and sent to the neuronavigator InVesalius.

Figure 2 depicts the experimental setup. The participant (the first author: 29-year-old man with no known neurological disorders or visible facial deformations) was instructed to sit in a chair and to stay relaxed and with a neutral facial expression. For neuronavigation, we used a T1-weighted magnetic resonance imaging acquired with a volumetric gradient echo sequence (voxel size 1 × 1 × 1 mm^3^; 240 × 240 × 240 acquisition matrix) in an Achieva 3 T scanner (Philips Healthcare, Netherlands). A figure-of-eight TMS coil (Neurosoft, Russia) was placed over the scalp directly above the left hand knob of the primary motor cortex and the scalp coordinate set as the target in InVesalius. The coil was oriented approximately perpendicular to the central sulcus. The revisiting experimental procedure included three steps: (1) coregistration between *MarLe* and the secondary tracker device, (2) neuronavigation coregistration, and (3) repositioning of the TMS coil. For the coregistration of the trackers, we collected 500 paired poses. The neuronavigation coregistration was performed using three fiducial landmarks: nasion, right and left ear tragus [3]. The TMS coil was initially placed on a side table, which was defined as the home position. The coil was repositioned 10 times, alternating between the home and the scalp target. The InVesalius Guiding interface was used to place the coil at the target and the coil coordinates were saved when the user reached the target within a 3-mm and 3° range. The experimental procedure was repeated three times for each of the three cameras. The experimental procedures followed our previous characterization protocol employed for the InVesalius Navigator [3].

### Statistical analysis

The stability was evaluated as the standard deviation (SD) of the difference of the translation (Euclidean distance) and three orientation vectors from the average pose coordinates during the acquisition. The effect of camera model, the camera–face distance, and the presence of filter were assessed with a two-way analysis of variance (ANOVA). The jittering of translation axis and rotation angles were evaluated with one-way ANOVA. The accuracy for revisiting a TMS coil repositioning was estimated as the average Euclidian distance and angles difference between the acquired coil pose and the predefined coil target. We used two-way ANOVA to evaluate if the camera model and the coordinates axes (translation, yaw, pitch, and roll) affects the revisiting target accuracy. Tukey HSD post-hoc multiple comparisons were performed for stability, jittering, and repeatability of coil positioning; The threshold for statistical significance was set at *p* < 0.05. The software R 4.2 (R Core Team, Austria) was used for all statistical analysis.

## Results

### MarLe stability and jittering

The measured stability for each distance, presence of filter, and for all tested cameras are depicted in Table 1. We observed that the stability, with filtering, varied across the camera model (${F}_{\text{2,16191}}=7.88$; *p* < 0.001). The c270’s average standard deviation was 0.02 mm (*p* < 0.001) smaller than the VCU Vega VT and 0.01 mm (*p* = 0.02) higher than the camera c920. The average standard deviation for the non-filtered coordinates was 68 ± 5% higher than the filtered coordinates for the camera C270, 70 ± 2% higher for the camera C920 and 37 ± 9% higher for the built-in camera Vega VT. The filtered coordinates did not reveal relevant differences between the camera–face distances (${F}_{\text{2,16191}}=0.94$; *p* = 0.39). The unfiltered coordinates did not reveal relevant differences between the cameras (${F}_{\text{2,16191}}=2.22$; *p* = 0.11) or for the camera–face distances (${F}_{\text{2,16191}}=0.10$; *p* = 0.90). No difference was found between the three trials (*p* = 0.99).

**Table 1:** The stability results for the distance between the camera and face, for the camera model, with and without filtering for each translation and rotation axis

Camera	Distance	± SD X (mm)	± SD Y (mm)	± SD Z (mm)	± SD Yaw (°)	± SD Pitch (°)	± SD Roll (°)
		with / without filter	with / without filter	with / without filter	with / without filter	with / without filter	with / without filter
**C270**	100 cm	0.14 / 0.37	0.10 / 0.23	0.36 / 1.22	0.09 / 0.34	0.11 / 0.37	0.04 / 0.14
	125 cm	0.14 / 0.37	0.10 / 0.23	0.36 / 1.22	0.09 / 0.34	0.11 / 0.37	0.04 / 0.14
	150 cm	0.13 / 0.43	0.08 / 0.22	0.39 / 1.14	0.10 / 0.30	0.14 / 0.51	0.04 / 0.13
**C920**	100 cm	0.07 / 0.25	0.07 / 0.19	0.28 / 0.90	0.08 / 0.27	0.10 / 0.32	0.03 / 0.10
	125 cm	0.09 / 0.31	0.07 / 0.22	0.41 / 1.37	0.10 / 0.37	0.10 / 0.32	0.03 / 0.11
	150 cm	0.10 / 0.35	0.07 / 0.22	0.31 / 1.06	0.08 / 0.27	0.14 / 0.45	0.03 / 0.12
**VCU** **Vega VT**	100 cm	0.16 / 0.27	0.12 / 0.18	0.62 / 0.87	0.26 / 0.32	0.16 / 0.27	0.07 / 0.12
	125 cm	0.18 / 0.32	0.14 / 0.23	0.91 / 1.31	0.32 / 0.41	0.17 / 0.32	0.08 / 0.14
	150 cm	0.18 / 0.34	0.15 / 0.23	0.85 / 1.24	0.28 / 0.37	0.22 / 0.41	0.08 / 0.14

**Fig. 3:**
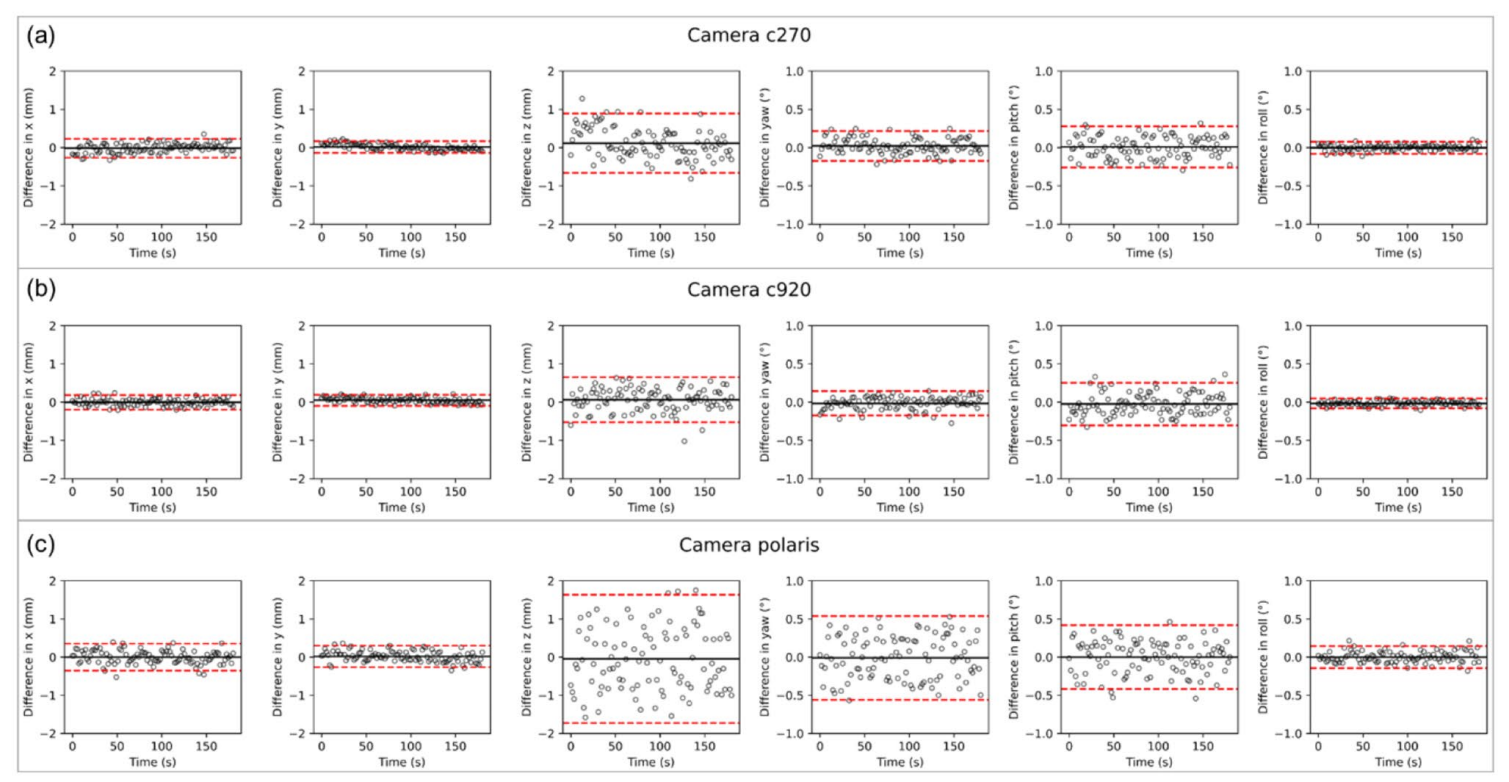
Jittering was evaluated for the translation axes (*x*, *y*, *y*) and rotation angles (yaw, pitch, roll) of three cameras, namely c270 in **a**, c920 in **b**, and VCU Polaris Vega VT in **c**. Data points were sampled every 2 s for 180 s, from a frontal human face photo. The black solid line is the average, and the red dashed lines are the 95% intervals (1.96 times the standard deviation)

*MarLe* jittering was estimated with a camera–head distance of 100 cm; the results are illustrated in Fig. 3. The charts points (black circles) are the distances between the measured head pose to the average head pose coordinate, recorded along 180 s for each translation axis (*x*, *y*, *z*) and rotation angle (yaw, pitch, roll). The red dashed lines represent the jittering, i.e., the 95% intervals (1.96 times the standard deviation) of the static head pose estimation. The camera c270 has a significant difference between the translation axis (Fig. 3a; *p* < 0.001; ${F}_{\text{2,897}}=21.30$). The jittering for the *x* axis is 0.11 mm smaller than for the *y* axis (Fig. 3a; *p* < 0.001) and 0.14 mm smaller than the *z* axis (Fig. 3a; *p* < 0.001). No difference was found for the rotation angles (Fig. 3a; *p* = 0.71; ${F}_{\text{2,897}}=0.34$). We did not find any difference for the translation axis and rotation angles for the cameras c920 (Fig. 3b; translation: *p* = 0.84; ${F}_{\text{2,897}}=0.18$; rotation: *p* = 0.39; ${F}_{\text{2,897}}=0.94$) and VCU Vega VT (Fig. 3c; translation: *p* = 0.19; ${F}_{\text{2,897}}=1.67$; rotation: *p* = 0.52; ${F}_{\text{2,897}}=0.61$). The smallest deviation was found for c920, ± 0.12 mm for the *y* axis and ± 0.06° for roll rotation, Fig. 3b. The largest deviation was ± 1.78 mm obtained for the *z* axis and ± 0.62° obtained for yaw rotation, both for the Vega VT camera, Fig. 3c. The 95% intervals were within the range of ± 2 mm and ± 1° for all cameras.

### Accuracy of revisiting a TMS target

**Fig. 4:**
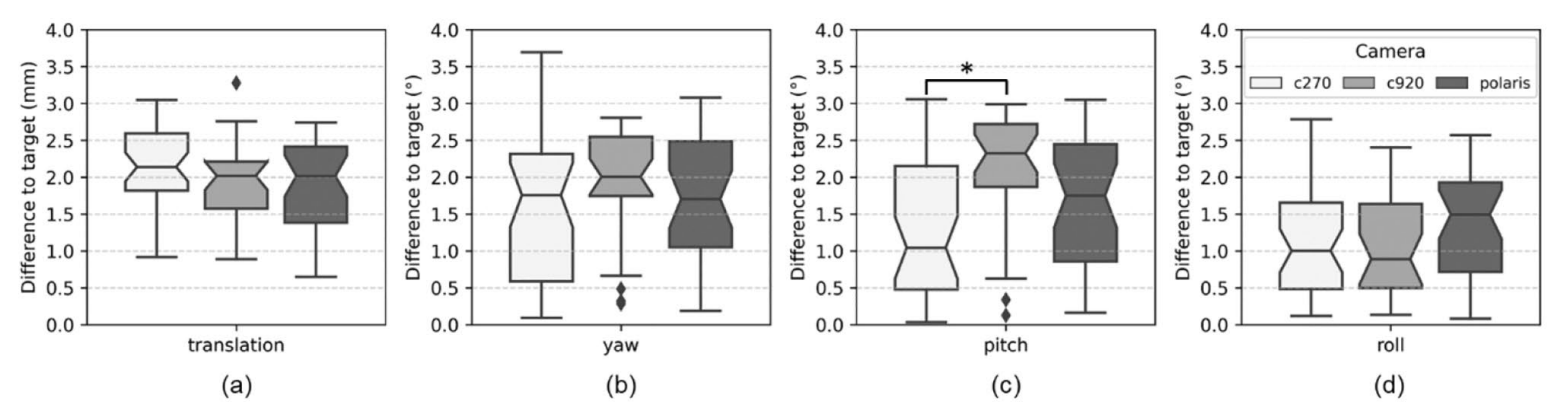
The difference to the target in translation in **a**, and rotation angles (yaw in **b**, pitch in **c**, roll in **d**) for cameras c270 (white boxes), c920 (light gray boxes), and VCU Polaris Vega VT (dark gray boxes) obtained in the mockup navigated TMS experiment. * *p* < = 0.001

We evaluated the accuracy of *MarLe* for revisiting a TMS coil placement in the mockup navigated TMS experiment. The coregistration error between the camera and the TMS coil tracker was 1.56 ± 0.96 mm for the built-in Vega VT, the low-cost camera c270 returned an error of 1.48 ± 1.10 mm and 1.38 ± 0.84 for the camera c920. The neuronavigation fiducial registration error was lower than 3 mm for all cameras and trials. The comparison of the translation vector and the rotation angles between the three cameras is ilustrated in Fig. 4. The difference between the collected coordinates to the target varied depending on the rotation angle (${F}_{\text{2,288}}=12.32$; *p* < 0.001) and the camera model (${F}_{\text{2,288}}=4.63$; *p* = 0.01). However, the camera model had no significant effect on the translation vector (Fig. 4a; ${F}_{\text{2,96}}=1.79$; *p* = 0.17). MarLe has a significant deviation of 0.43° between pitch and roll (Fig. 4c and d; *p* < 0.001), 0.55° between yaw and roll (Fig. 4b and d; *p* < 0.001). The cameras c270 and c920 have a significant deviation of 0.86° in pitch (Fig. 4c; *p* = 0.002).

## Discussion

During conventional navigated TMS, the markers or sensors fixed on the patient’s head are susceptible to displacements. Uncontrollable factors, such as patients sweating or oily skin can cause the markers to move from the initial fixed position or the skin may move due to head muscle activation, leading to neuronavigation inaccuracies. Neuronavigation systems cannot distinguish head marker displacements; the mismatch detection depends on the user’s expertise [39].

To overcome these limitations, we developed a markerless head tracking algorithm, named *MarLe*, for navigated TMS that can estimate the head pose based on real-time video processing. We combined, in a single device, the optimal accuracy of infrared marker tracking for the TMS coil with the *MarLe* based on the built-in video camera. Nevertheless, we can use standalone low-cost cameras to perform the head-pose estimation. In a simulated TMS experiment, neuronavigation with *MarLe* achieves acceptable accuracy and stability with the built-in Vega VT camera and with two low-cost webcams, comparable to dedicated tracking devices supported by InVesalius [3].

The camera calibration errors were below 1 mm for all cameras. Camera calibration is a critical component in performing accurate tracking [31]. Our *MarLe* algorithm estimates the head pose by relating the camera units (pixels) to the physical world (meters) based on the camera calibration parameters. Therefore, the camera calibration error affects the navigation accuracy. The neuronavigation error is mainly due to the coregistration variability, tracking device inaccuracy, and distortions in the anatomical images [40]. The typically recommended error limits for neuronavigation systems are 3 mm and 3° for positional and orientation accuracy, respectively, i.e., the inherent error of a neuronavigation system to reach a target [4]. Some studies define the neuronavigation error based on the 95th percentile distribution of Euclidean distances between the target and the collected coordinate. In this approach, the accepted neuronavigation error is up to 3–4 mm [3, 41]. The stability and jittering experiments enabled the assessment of the tracking device accuracy while revisiting a TMS coil placements assessed the overall neuronavigation error with *MarLe*.

Our measurements revealed that the distance between camera and face, a distance ranging from 100 to 150 cm, did not affect the *MarLe* stability to estimate head pose. One likely explanation is that we are operating in the camera’s optimal measurement volume. The *MarLe* operational distance range provides setup flexibility for the users, reducing the time spent with tracker arrangement for navigated TMS. The stability *MarLe*, for filtered coordinates, depends on the resolution and acquisition frame of the camera. VCU Vega VT has the highest resolution (2048 × 1536 pixels), followed by c920 (1920 × 1080), with c270 having the lowest resolution (1280 × 720); the resolution affects the accuracy of head-pose determination [31]. Higher resolution means smaller pixel size, providing a better capability to detect small head movements. *MarLe* head-pose estimation stability seems to be the same for cameras with resolutions of 2048 × 1536 pixels and 1920 × 1080 pixels, but a lower resolution may affect the *MarLe* stability.

The stability for the non-filtered coordinates is not affected by the camera device or the camera–face distance. Then, filtering can increase stability, but this depends on frame rate. Namely, the Savitzky–Golay filter is affected by the amount of input data over time [42]. A high acquisition rate results in a stronger filter effect, increasing the smoothing and stability of the head–pose estimation. However, the resolution seems to have no effect on the filtering. For example, the built-in camera Vega VT has the lowest frame rate (20 Hz), and it has the smallest increase in stability (37 ± 9%) after filtering. The cameras c270 and c920 have a 30-Hz acquisition rate; both have similar stability increases after filtering, 68 ± 5% and 70 ± 2%, respectively. The Savitzky–Golay filter has an important contribution to increasing the stability and consequently the accuracy of *MarLe*. It should be noted that the Savitzky–Golay filter parameters were optimized to have the lowest delay in terms of smoothness. The first-order polynomial is used due to the good response, in terms of smoothness, for low frequencies (less than 100 Hz) [43]. The window size affects the time response; a big window size includes delays to filtered signals. We found five frames to be optimal: there was no noticeable visual delay, and a good filter response was obtained.

The *MarLe* jittering for all cameras was less than 2 mm and 1° for translation and rotation, i.e., lower than the acceptance limit of 3 mm or 3°. Interestingly, fluctuations when using the *MarLe* algorithm are clearly smaller than those obtained with other head-pose estimation algorithms published up to now [24, 44]. Martins and Batista[24] found a jitter of 1 cm and 2°. This discrepancy may be caused by the face-detection algorithm. Martins and Batista[24] used a statistical matching method (active appearance mode) to track facial characteristics. We are using a pre-trained network based on the 300-W face dataset to perform facial landmark detection [34]. This dataset has 300 indoor and 300 outdoor images including a broad range of age, sex, facial expressions, face sizes, face shapes, skin tones, illumination conditions, and occlusions, which may provide an improved performance of markerless navigation on varying room conditions and distinct facial characteristics [34].

The *z* translation axis has the higher deviation, illustrated in Fig. 3. The *z* direction defines the distance between the source, i.e., the camera, to the tracked object, i.e., the face. The head-pose estimation algorithm is based on a face model; once the algorithm detects a face, we use a closed-form solution to find the required scaling to fit the face model to the detected face. The translation *z* is based on the scaling factor, and it is more susceptible to fluctuations than the *x* and *y* axes [31]. However, no significant difference was found between the translation axes (*x*, *y*, *z*) and between the rotation angles (yaw, pitch, roll) for the c920 and built-in Vega VT. The camera c270 has a deviation only between the translation axes. This might be caused by the low camera resolution [31].

Finally, the *MarLe* accuracy for revisiting a coil placement was in a range of 3–4 mm and 3–4° for the 95th percentile, corroborating previous findings [3, 41]. As was expected, *MarLe* connected to c270 showed the highest deviations for revisiting a TMS target. Again, this association might point toward low camera resolution [31].

### Limitations

It is important to highlight that the TMS coil tracker and the camera must be stationary over the TMS session. The transformation between the camera to the coil tracker is based on the physical location of the devices. Displacements will nullify the transformation matrix and the coregistration must be redone. However, we could overcome this limitation with the Polaris Vega VT.

We should note that *MarLe* characterization and the simulated TMS experiment were performed with neutral facial expressions in a well-illuminated room, which is the most common condition on a navigated TMS experiment with healthy participants. The facial landmark detection method implemented in *MarLe* has excellent performance for a variety of facial characteristics [34]. However, in this study, the specific effect of varying conditions, such as skin tone, sex, and age, were not characterized in terms of TMS targeting accuracy and stability, which are planned to be explored in a future study. Furthermore, outdoor scenes with poor illumination and the facial expressions of, e.g., surprise and scream, can negatively impact the performance of data-driven face detection methods [34]. For *MarLe*, we visually inspected that the facial detection stability decreases when the participant smiles or talks. One possible solution is to combine facial expression recognition with face detection to warn the user when a non-neutral expression is identified, avoiding unstable pose estimations. One might also be able to teach the algorithm to use facial landmarks that do not move when facial expressions change, improving stability and accuracy in dynamic conditions. Lastly, we did not test our method on participants with facial deformities. The heterogeneity of facial deformities may impose difficulties in obtaining accurate and generalizable landmark detection, which may provide an important topic to be investigated [45].

## Conclusion

We developed a markerless head-pose estimation algorithm for navigated TMS that utilizes low-cost cameras. We successfully demonstrated the registration of the neuronavigation system, tracking device, and camera used for head-pose estimation through transformation matrixes. *MarLe* has a potential to improve the reliability and ease of TMS targeting, simplifying, and reducing the time to perform the determination of head pose, as there is no need to be concerned about the displacement of head-tracker markers.

## Electronic supplementary material

Supplementary Material

## Abbreviations

ANOVA: Analysis of variance
FPS: Frames per second
nTMS: Navigated transcranial magnetic stimulation
SD: Standard deviation
3D: Three-dimensional
TMS: Transcranial magnetic stimulation
2D: Two-dimensional
VCU: Video camera unit
