# Supplementary material for: MarLe: Markerless estimation of head pose for navigated transcranial magnetic stimulation
Source: Phys Eng Sci Med. 2023 May 11;46(2):887–96. doi: 10.1007/s13246-023-01263-2 (PMC10209239; doi:10.1007/s13246-023-01263-2)
Supplement: Supplementary file 1 — Supplementary Material [file 13246_2023_1263_MOESM1_ESM.doc]

**Supplementary Material**

***MarLe*: Markerless estimation of head pose for navigated transcranial magnetic stimulation**

Renan H. Matsuda^1,2*^, Victor Hugo Souza^1,2,3^, Petrus N. Kirsten^1^, Risto J. Ilmoniemi^2^, Oswaldo Baffa^1^

^1^ Department of Physics, Faculty of Philosophy Sciences and Letters of Ribeirão Preto, University of São Paulo, Av. Bandeirantes, 3900, 14040-901 Ribeirão Preto – SP, Brazil.

^2^ Department of Neuroscience and Biomedical Engineering, Aalto University School of Science, Rakentajanaukio 2, 02150 Espoo, Finland.

^3^ School of Physiotherapy, Federal University of Juiz de Fora, Cascatinha, Juiz de Fora ¬– MG, Brazil.

*Corresponding author:

Department of Physics

Faculty of Philosophy Sciences and Letters of Ribeirao Preto

University of São Paulo

Av. Bandeirantes, 3900, CEP: 14040-901, Monte Alegre, Ribeirão Preto, Brazil

E-mail: renan.matsuda@usp.br

**Evaluation of jittering filters**

In this study, we examined the effectiveness of three distinct jittering filters: the Kalman filter [1], Grubbs filter [2], and Savitzky-Golay filter [3]. To assess the performance of each filter, we conducted experiments using a static face image captured with a camera c920 positioned at 100 cm from the subject's head. We recorded position (x, y, and z) and orientation (yaw, pitch, and roll) data over a three-minute period and estimated the position vector (PV) and orientation vector (OV) for each frame using the equations outlined below.

$$PV= \sqrt{x^{2}+y^{2}+z^{2}}$$

$$OV= \sqrt{{yaw}^{2}+{pitch}^{2}+{roll}^{2}}$$

To evaluate the stability of the pose coordinates obtained using each filter, we calculated the standard deviation of the position and orientation vectors. We estimated the jittering attenuation rate based on the following equations:

$${Attenuation}_{position}= 10*\log_{10} \left( \frac{{PV}_{no filter}}{{PV}_{filtered}} \right)$$

$${Attenuation}_{orientation}= 10*\log_{10} \left( \frac{{OV}_{no filter}}{{OV}_{filtered}} \right)$$

The resulting stabilities for each filter are presented in Table S1. Additionally, the jittering over time and the jittering boxplot are depicted in Fig. S1 and Fig. S2, respectively.

**Table S1** The stability and the jittering attenuation rate results for each evaluated filter for the position and orientation vector.

| Filters | Stability Position (mm) | Position jittering attenuation (dB) | Stability Orientation (°) | Orientation jittering attenuation (dB) |
| --- | --- | --- | --- | --- |
| No filter | 0.81 | 0.00 | 0.28 | 0.00 |
| Grubbs | 0.69 | 0.68 | 0.24 | 0.70 |
| Kalman | 0.39 | 3.16 | 0.14 | 3.18 |
| Savitzky-Golay | 0.28 | 4.55 | 0.01 | 4.52 |


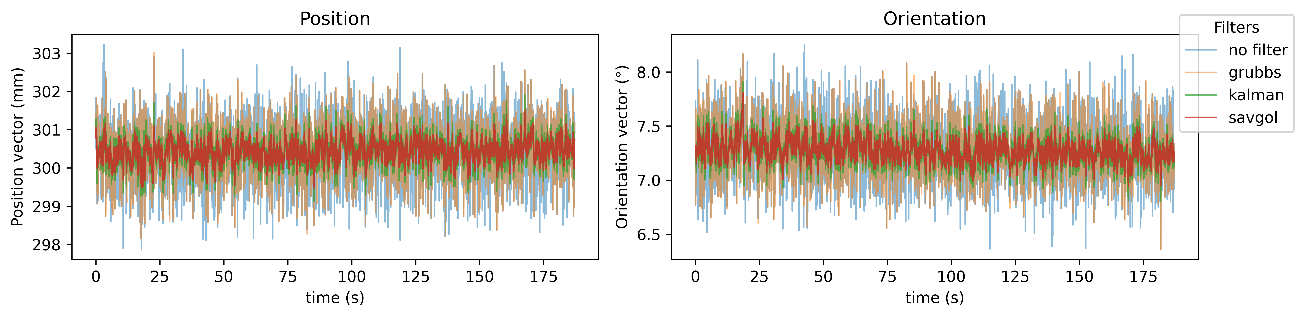


**Fig. S1** Jittering graph plot for the position and orientation vectors for the three evaluated filters, Grubbs, Kalman and Savitzky-Golay, and the unfiltered jittering, over a 3-minute acquisition


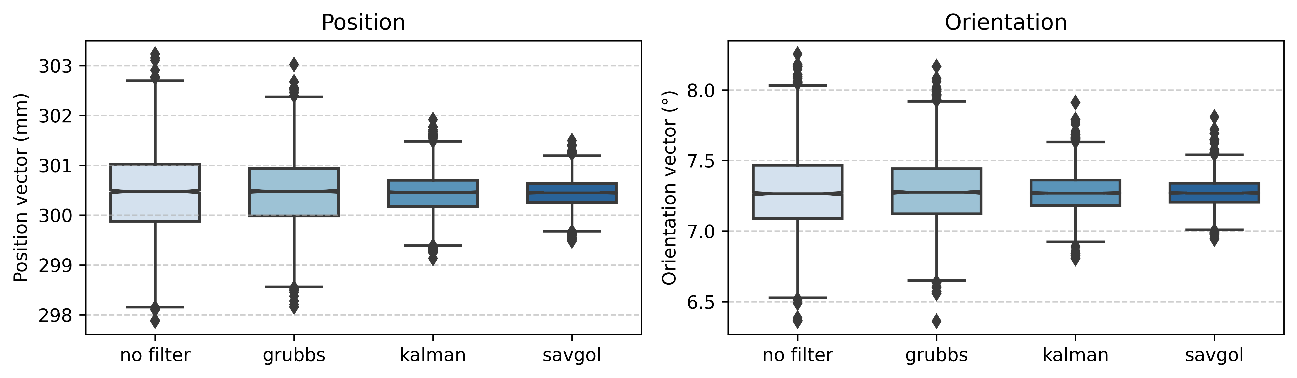


**Fig. S2** Boxplots for the position and orientation vector for the three evaluated filters, Grubbs, Kalman and Savitzky-Golay, and the unfiltered jittering

We employed a critical value of 1.42 and a window size of 4 samples for the Grubbs filter. For the Kalman filter, we set the process covariance at 0.01 and used a window size of 5 samples. Finally, for the Savitzky-Golay filter, we selected a window size of 5 samples. We optimized all filter parameters based on the filter response to ensure no significant visual delay was observed.

After comparing the results obtained from each filter, we found that the Savitzky-Golay filter was the most effective for *MarLe*. While the Kalman filter also demonstrated promising performance, we ultimately selected the Savitzky-Golay filter due to its slightly superior results as indicated by the smaller interquartile ranges in the boxplots of Fig. S2.

References

1. Kalman RE (1960) A new approach to linear filtering and prediction problems. Journal of Fluids Engineering, Transactions of the ASME 82:35–45. https://doi.org/10.1115/1.3662552

2. Grubbs FE (1969) Procedures for Detecting Outlying Observations in Samples. Technometrics 11:1–21. https://doi.org/10.1080/00401706.1969.10490657

3. Savitzky A, Golay MJE (1964) Smoothing and Differentiation of Data by Simplified Least Squares Procedures. Anal Chem 36:1627–1639. https://doi.org/10.1021/ac60214a047
